# Insights into Genetic Diversity, Runs of Homozygosity and Heterozygosity-Rich Regions in Maremmana Semi-Feral Cattle Using Pedigree and Genomic Data

**DOI:** 10.3390/ani10122285

**Published:** 2020-12-03

**Authors:** Filippo Biscarini, Salvatore Mastrangelo, Gennaro Catillo, Gabriele Senczuk, Roberta Ciampolini

**Affiliations:** 1 CNR-IBBA (National Research Council, Institute of Agricultural Biology and Biotechnology), 20133 Milan, Italy; 2 Dipartimento Scienze Agrarie, Alimentari e Forestali, University of Palermo, 90128 Palermo, Italy; salvatore.mastrangelo@unipa.it; 3 CREA Research Centre for Animal Production and Acquaculture, CREA, 00015 Monterotondo, Italy; gennaro.catillo@crea.gov.it; 4 Dipartimento di Agricoltura, Ambiente e Alimenti, University of Molise, 86100 Campobasso, Italy; g.senczuk@unimol.it; 5 Dipartimento di Scienze Veterinarie—Università di Pisa, 56124 Pisa, Italy; roberta.ciampolini@unipi.it

**Keywords:** maremmana cattle, runs of homozygosity, inbreeding, heterozygosity-rich regions, pedigree relationships, genomic relationships

## Abstract

**Simple Summary:**

In this study, we estimated genetic diversity in semi-feral Maremmana cattle using both pedigree- and genomic-based approaches, and detected regions of homozygosity (ROH) and heterozygosity (ROHet) in the genome, which are still poorly characterized in the Maremmana breed. A sensitivity analysis on the parameters used to detect ROH and ROHet provided information which can be useful to guide studies on the detection of genomic runs in general, and in semi-feral cattle populations in particular. The average observed and expected heterozygosity were estimated at $H_{O}=0.274$ and $H_{E}=0.261$, respectively. Pedigree-based average inbreeding (*F*) was estimated at 4.9%. A total of 3332 ROH and 1471 ROHet were detected in the genomes of the 149 animals included in the study. Genes found to be within the identified ROH and ROHet islands (e.g., *KCTD8*, *GNPDA2*) point to phenotypic characteristics related to environmental adaptation and robustness of Maremmana cattle. These results are of important because they will help design and implement breeding and conservation strategies for Maremmana cattle, and provide guidelines for other local cattle breeds.

**Abstract:**

Semi-feral local livestock populations, like Maremmana cattle, are the object of renewed interest for the conservation of biological diversity and the preservation and exploitation of unique and potentially relevant genetic material. The aim of this study was to estimate genetic diversity parameters in semi-feral Maremmana cattle using both pedigree- and genomic-based approaches ($F_{IS}$ and $F_{ROH}$), and to detect regions of homozygosity (ROH) and heterozygosity (ROHet) in the genome. The average heterozygosity estimates were in the range reported for other cattle breeds ($H_{E}=0.261$, $H_{O}=0.274$). Pedigree-based average inbreeding (*F*) was estimated at 4.9%. The correlation was low between *F* and genomic-based approaches (r=0.03 with $F_{IS}$, r=0.21 with $F_{ROH}$), while it was higher between $F_{IS}$ and $F_{ROH}$ (r=0.78). The low correlation between *F* and $F_{ROH}$ coefficients may be the result of the limited pedigree depth available for the animals involved in this study. The ROH islands identified in Maremmana cattle included candidate genes associated with climate adaptation, carcass traits or the regulation of body weight, fat and energy metabolism. The ROHet islands contained candidate genes associated with nematode resistance and reproduction traits in livestock. The results of this study confirm that genome-based measures like $F_{ROH}$ may be useful estimators of individual autozygosity, and may provide insights on pedigree-based inbreeding estimates in cases when animals’ pedigree data are unavailable, thus providing a more detailed picture of the genetic diversity.

## 1. Introduction

Semi-feral local livestock populations are an object of renewed interest for the conservation of biological diversity and the preservation and exploitation of unique and potentially relevant genetic material [1,2]. Basic preliminary steps for any conservation or exploitation programmes include the estimation of fundamental genetic diversity parameters that allow researchers and geneticists to characterize the population, assess the risk of loss of diversity and suggest the most appropriate management strategies (e.g., mating and selection schemes). Fundamental genetic diversity parameters to be estimated include the inbreeding coefficient of individual animals, the genetic relationships between animals, the overall levels of homozygosity and heterozygosity in the genome and their distribution along the chromosomes. Alongside traditional methods based on pedigree data, in recent years, methods based on genomic data have been developed for the estimation of genetic parameters. The availability of high-throughput sequencing or genotyping single nucleotide polymorphisms (SNP) data has opened up the possibility of characterizing individual segments of the genome in terms of their homozygosity (regions of homozygosity (ROH)) or heterozygosity (heterozygosity-rich regions). ROH are contiguous segments of homozygous genotypes that are present in an individual due to parents transmitting identical haplotypes to their offspring [3]. ROH have been widely used as predictors of whole-genome inbreeding levels (e.g., [4,5,6,7]), and the strong correlation between ROH-based estimates and genealogical inbreeding coefficients [8,9] suggests that in absence of pedigree data, ROH may be used effectively even from relatively few samples. The use of ROHs allows us also to distinguish between recent and ancient inbreeding [10]. Shared ROH within a population can be used to identify genomic regions potentially under selection, which may be involved in defining breed-specific traits or the adaptation to the environment or production systems (e.g., [11,12,13,14,15]). Heterozygosity-rich regions emerged as a more recent concept [16], and have been characterized by very few studies in animals (e.g., [17]).

Despite such advancements, the merit of genomic-based relative to pedigree-based methods has not been fully explored under all scenarios, particularly those related to local and/or marginal livestock populations. In addition, the sensitivity of ROH detection to the parameters used in the analysis (e.g., ROH length, SNP density, sliding-window-related parameters, etc.) is still unclear: so far only a handful of works addressed this issue (e.g., [18]), limited to some parameters and a restricted portion of the dimensionality space. All the above is especially relevant for small-sized local populations, where pedigree data are more likely to be missing and genomic tools are most promising to fill the gap to their full genetic characterization.

The aim of this study was to estimate genetic diversity parameters in semi-feral Maremmana cattle using both pedigree- and genomic-based approaches, and to detect regions of homozygosity and heterozygosity in the genome, which are still poorly characterized in the Maremmana breed. A sensitivity analysis on the parameters used to detect ROH and heterozygosity-rich regions provided information which can be useful to guide studies on the detection of genomic runs in general, and in semi-feral cattle populations in particular.

## 2. Materials and Methods

### 2.1. Samples, Genotyping and Data Editing

SNP genotype data (Illumina BovineSNP50 v2 BeadChip assay (Illumina Inc., San Diego, CA, USA)) for 149 heads of Maremmana cattle (138 females and 11 males) from two farms in central Italy (Alberese: *N* = 25; Tormancina: *N* = 124) were obtained from previously published studies [19,20]. These two sets of SNP genotypes were merged using PLINK v.1.9 [21], yielding a total of 54571 SNPs. SNP positions (chromosome and basepairs) were updated using the Bos taurus ARS-UCD1.2 (bosTau9) genome assembly. Markers were filtered to exclude loci assigned to unmapped contigs and to sex chromosomes. Additional quality control included call-rate ≥ 0.95 for SNP loci. SNPs that did not satisfy these quality criteria were excluded. SNP loci were not filtered out for low MAF, since this is a single breed study, and we wanted to be able to properly detect homozygosity [9,18]. Animals with more than 15% missing SNPs were also removed from further analyses.

Since SNP data were obtained from previous studies, no specific ethical approval was required. No actual experimental research on animals nor sampling were performed.

### 2.2. Genetic Diversity Coefficients

PLINK v.1.9 was used to estimate basic genetic diversity indices including: observed and expected heterozygosity ($H_{O}$ and $H_{E}$, respectively), minor allele frequency (MAF) and the genomic inbreeding coefficient, on the basis of the difference between the observed and expected numbers of homozygous genotypes ($F_{IS}$). The contemporary effective population size (Ne) was estimated using NEESTIMATOR v.2 [22] according to the random mating model of the LD method. We used a method described by Waples and Do [23] to add a bias correction to the original LD method and a threshold of 0.05 as the lowest allele frequency to derive the least biased results. We reported our estimates with 95% confidence intervals.

### 2.3. Runs of Homozygosity Detection and Distribution

ROH was identified in every individual using PLINK v1.9. A sliding-window approach was used to scan individual SNP genotypes and detect homozygous segments. The parameters applied to define an ROH were: (i) a sliding window of 50 SNPs across the genome; (ii) the proportion of homozygous overlapping windows was 0.05 (this number is relatively low, so that SNPs at the edge of a true segment will be called, as long as the windows are sufficiently large, such that the probability of a window being homozygous by chance is sufficiently small); (iii) the minimum number of consecutive SNPs included in an ROH was 30; (iv) the minimum length of an ROH was set to 2 MB; (v) the maximum gap between consecutive homozygous SNPs was 1000 kB; (vi) a density of one SNP per 100 kB; and (vii) a maximum of one SNP with missing genotype and up to one heterozygous genotype were allowed in an ROH. Considering that high linkage disequilibrium (LD) can lead to the detection of ROH that are more likely to be IBS (identical by state) rather than IBD (identical by descent), we considered ROH > 2 MB in length to exclude short common ROH. Moreover, we allowed one heterozygous SNP inside ROH to avoid underdetection of long ROH. In fact, not allowing heterozygous SNP genotypes to be present in a homozygosity run, as commonly done in human genetics, is not adequate for livestock populations because they have much higher levels of autozygosity and therefore longer ROH. Furthermore, since genotyping errors in SNP chip data can occur, it seems more reasonable to allow for one heterozygous call. The mean number of ROH and the average length of ROH per animal were calculated. ROH were divided into seven classes (2 to 4, 4 to 8, 8 to 12, 12 to 16, 16 to 20, 20 to 30 and > 30 MB). The number and percentage of ROH within each ROH length category were determined.

From ROH, individual inbreeding coefficients ($F_{ROH}$) were estimated as the ratio between the sum of the length of all ROH ($L_{ROH}$) and the total length of the autosomal genome covered by SNPs on the array ($L_{AUTO}$ = 2541 MB). Based on ROH length, recent and ancient inbreeding can be also estimated [24,25]: the longer the ROH, the more recent the inbreeding. For this purpose, ROH were grouped by length: 2–4 MB, 4–8 MB, 8–16 MB, longer than 16 MB. From each ROH-class, $F_{ROH}$ was also calculated as the ratio between the total ROH length (in that class, e.g., $L_{ROH2-4}$) and $L_{AUTO}$. To identify the genomic regions that were most commonly associated with ROH, the percentage of occurrences of SNP in ROH was estimated by counting the number of times that each SNP appeared in a ROH and dividing that number by the number of animals, allowing us to obtain a locus homozygosity range (from 0 to 1). To identify highly homozygous genomic regions (ROH islands), SNPs from the 99.9 quantiles of the locus homozygosity (SNP inside ROHs) distribution were selected; this translated to an in-ROH frequency of 0.41 as the threshold for SNPs to be included in the ROH islands.

### 2.4. Sensitivity Analysis

In literature, different criteria have been reported to define and identify ROH, and this is known to have an impact on results [18,26]. In this study, therefore, we wanted to test the sensitivity of our results to some of the parameters used for ROH detection. The parameters tested in this study were SNP density (from 1 SNP every 50 kB to 1 SNP every 100 kB) and the maximum number of heterozygous (from 0 to 5) and missing (from 0 to 5) genotypes allowed in an ROH. To evaluate the sensitivity of ROH detection results to different values of these parameters, the following variables were monitored: number of ROH detected, average number of ROH per animal, average ROH size and average ROH-based inbreeding ($F_{ROH}$).

### 2.5. Detection of Heterozygosity-Rich Regions

Heterozygosity-rich regions (a.k.a. “runs of heterozygosity”, ROHet, [16]) were detected using the *R* package detectRUNS [27] by applying the consecutive method [28]. The following parameters were used for the detection of ROHet: (i) the minimum number of consecutive SNPs included in a ROHet was 15; (ii) the minimum length of a ROHet was set to 250 kB; (iii) the maximum gap between consecutive homozygous SNPs was 1000 kB; (iv) a maximum of two SNP with missing genotype and up to three homozygous genotypes were allowed in a ROHet. Highly heterozygous genomic regions (“ROHet islands”) were identified by selecting SNPs with an in-ROHet frequency > 0.25.

### 2.6. Genealogical Analysis

Pedigree data for the animals from the two farms were available: the pedigree file contained 1439 records and included 119 sires and 512 dams, with average depth of 4.2 generations. The numerator relationship matrix (A) of additive genetic relationships between individuals was estimated from pedigree data [29]; the diagonal elements of matrix A are $1+F_{i}$, where $F_{i}$ are the inbreeding coefficients (probability of identical-by-descent (IBD) alleles at any given locus), with i∈[1,numberofanimals] [30]. The *R* package pedigree was used for pedigree calculations [31].

### 2.7. SNP-Based and ROH-Based Genomic Relationships

Genomic relationships between animals were estimated based on (i) SNP genotypes, and (ii) ROH. When genotypes were used directly, the method described by Van Raden [32] was applied: briefly, centered SNP genotypes were multiplied pairwise between individual animals and scaled by twice the sum of allele frequencies. For the estimation of genomic relationships à la Van Raden, missing SNP genotypes were previously imputed using the localized haplotype clustering imputation method implemented in the computer package “Beagle” v.4.1 [33]. The imputation of missing SNP genotypes was applied only for this analysis. To estimate ROH-based genomic relationships, the total length of ROH overlapping between individuals was calculated and then scaled by the sum of ROH frequency weighted by ROH length. In this way, longer ROH, which are more likely to be IBD, contributed proportionally more to the inter-individual relationship estimate. ROH results used to calculate the ROH-based relationship matrix were produced using the parameters described for the base scenario.

### 2.8. Gene Annotation

Information on the annotated genes within the highly homozygous (ROH islands) and heterozygous (ROHet islands) genomic regions detected in Maremmana cattle were obtained from the Genome Data Viewer tool provided by NCBI (https://www.ncbi.nlm.nih.gov/genome/gdv/browser/genome/?id=GCF_002263795.1). The *Bos taurus* genome assembly ARS-UCD1.2 was used as reference.

## 3. Results

### 3.1. Genetic Diversity

After filtering for quality, the final number of SNPs retained for the analysis was 49,086. All animals had high quality genotyping and were included in the analysis. The average observed ($H_{O}$) and expected ($H_{E}$) heterozygosity estimates were 0.274±0.204 and 0.261±0.189, respectively, and the average MAF was 0.195±0.163. The estimated contemporary effective population size (Ne) in Maremmana cattle was 23.5.

### 3.2. Pedigree and Genomic Relationships and Inbreeding Coefficients

**Figure 1:**
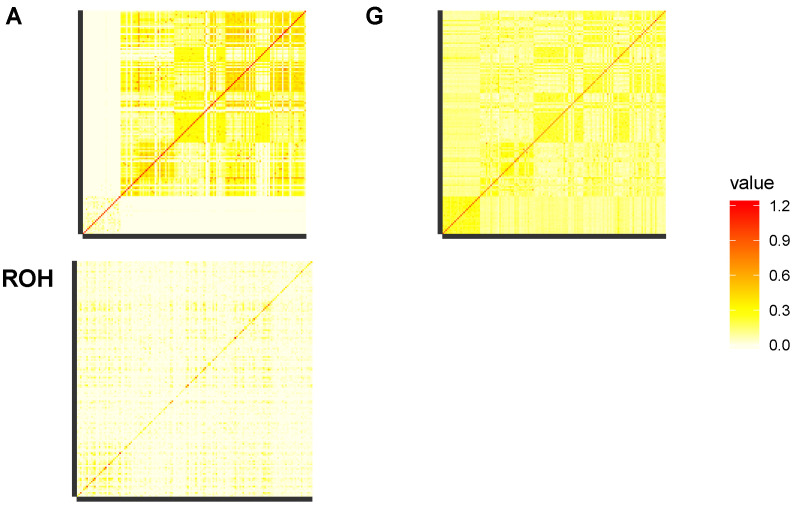
Matrix of genealogical ((A), **top left**), single nucleotide polymorphisms (SNP)-based ((G), **top right**) and regions of homozygosity (ROH)-based (**bottom left**) relationships between sampled individuals. Individuals are ordered by the farm they came from (Alberese, Tormancina).

**Figure 2:**
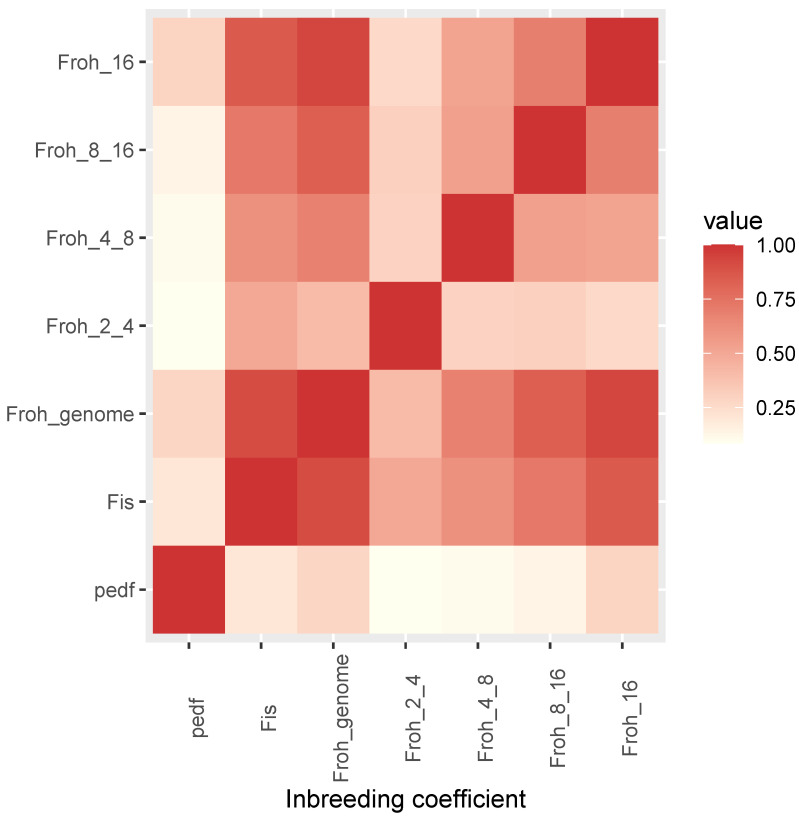
Heatmap of the correlation matrix between different measures of inbreeding: genealogical inbreeding (PedF), SNP-based genomic inbreeding (Fis), whole-genome ROH-based inbreeding (Froh), ancient (Froh based on short ROH: 2–4 MB, 4–8 MB) and recent (Froh based on long ROH: 8–16 MB, >16 MB) inbreeding.

Additive genetic relationships between animals were calculated based on: (i) pedigree (matrix A), (ii) SNP genotypes (matrix G), and (iii) ROH (matrix ROH). Heatmaps of the relationship matrices are shown in Figure 1. From all three sets of relationship data, inbreeding coefficients were estimated: from pedigree data, the average $F_{i}$ was F=0.049±0.062 (range [0,0.209]); from SNP genotypes the average $F_{IS}$ was −0.053±0.113 (range [−0.592,0.191]) (negative values correspond to cows with lower than average homozygosity); from the ROH detected with base-scenario parameters, the average $F_{ROH}$ ($F_{ROH>2\mathrm{MB}}$) was 0.077±0.055 (range [0.002,0.272]). Pearson correlations between inbreeding coefficients were $r(F,F_{IS})=0.027$, $r(F,F_{ROH})=0.207$, $r(F_{IS},F_{ROH})=0.780$. Spearman correlations were higher: $\rho (F,F_{IS})=0.201$, $\rho (F,F_{ROH})=0.279$, $\rho (F_{IS},F_{ROH})=0.911$. Inbreeding coefficients were also calculated from short ROH (ancient inbreeding) and long ROH (recent inbreeding). Figure 2 shows the heatmap of the correlation matrix between different estimates of inbreeding coefficients. Estimates of recent inbreeding form a correlation cluster ($\rho (F_{ROH8-16},F_{ROH16+})=0.690$, $\rho (F_{ROH4-8},F_{ROH8-16})=0.538$, $\rho (F_{ROH4-8},F_{ROH16+})=0.517$), while they show weaker correlations with estimates of more ancient inbreeding ($\rho (F_{ROH2-4},F_{ROH4-8})=0.293$, $\rho (F_{ROH2-4},F_{ROH8-16})=0.301$, $\rho (F_{ROH2-4},F_{ROH16+})=0.264$).

### 3.3. Runs of Homozygosity

From the base scenario, a total of 3332 ROH were identified in the 149 animals. Except two animals, all Maremmana cows had at least one ROH > 2 MB. The average number of ROH per animal was 22.27 with values ranging from 1 to 51. The average ROH length detected across all autosomes was 8.55 MB, with considerable variation: the shortest ROH was 2 MB found on BTA04 and the longest ROH was 75.57 MB on BTA03. Number of ROH, percentage of total, and average length per size class are reported in Table 1.

**Figure 3:**
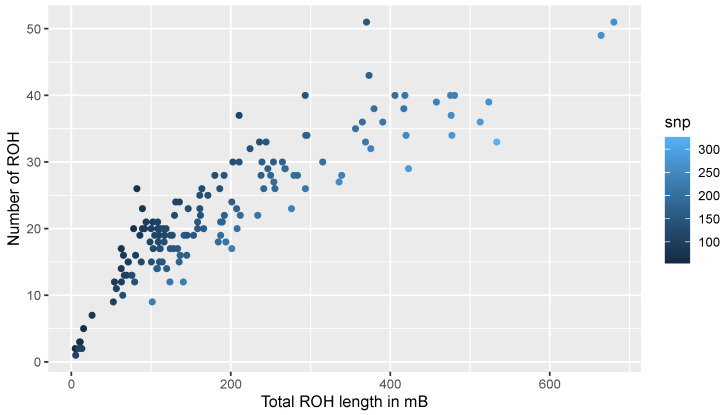
Total length of ROH vs number of ROH per animal in the Maremmana cattle dataset. ROH length is expressed in MB.

Figure 3 shows the relationship between the number of ROH segments and the length of the genome covered by ROH per individual. The majority of the individuals clustered close to the origin of coordinates because each one carried from 1 to 30 ROH with a total length < 200 MB. The two animals with the highest level of homozygosity showed 685.85 MB, and  670.09 MB of their genome classified as ROH.

### 3.4. Heterozygosity-Rich Regions

A total of 1471 ROHet were identified in Maremmana cattle using the chosen parameters. Most animals (110 out of 149) had between 3 and 9 ROHet in their genome. The average ROHet length detected across all autosomes was 701.54 kB: the shortest ROHet was 288.1 kB on BTA21 and the longest ROHet was 2109 kB on BTA11. Number of ROHet, percentage of total, and average length per size class are reported in Table 2.

### 3.5. Runs of Homozygosity (ROH) and Heterozygosity (ROHet) Islands

**Figure 4:**
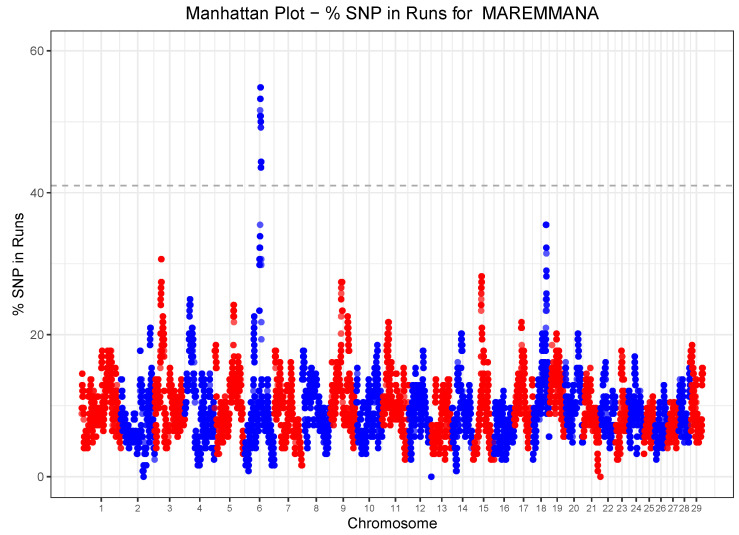
Manhattan plot of the proportion of times each SNP falls within a ROH in the analyzed Maremmana cattle population. The dashed horizontal line is the 41% threshold obtained from the SNP homozygosity distribution.

**Figure 5:**
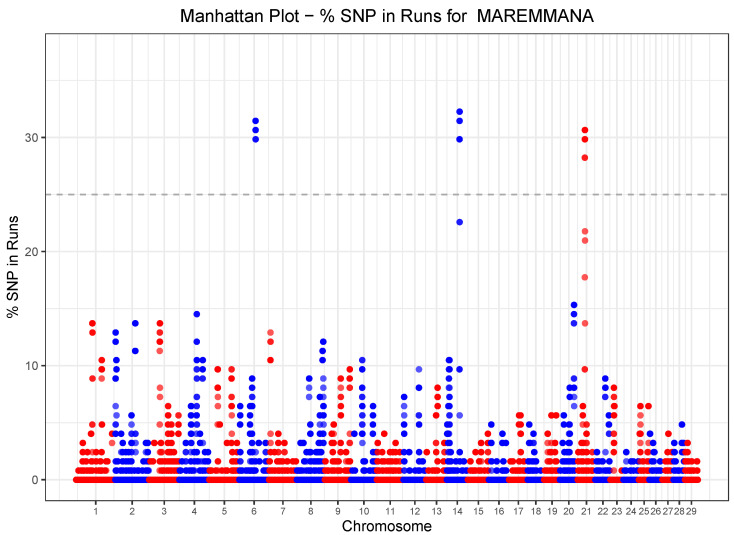
Manhattan plot of the proportion of times each SNP falls within a ROHet (heterozygosity-rich region) in the analysed Maremmana cattle population. The dashed horizontal line is the 25% threshold.

ROH islands were identified by taking the 99.9 quantile of the SNP-inside-ROH frequency distribution, which corresponds to a frequency threshold of 0.41 (Figure 4). Only one genomic region of about 3 MB on BTA06, with 58 SNPs, was detected as a ROH island, which harbored a total of six genes with known function (Table 3). Based on a SNP-inside-ROHet frequency > 0.25 three ROHet islands were identified in the genome of Maremmana cattle (Figure 5). Table 3 provides the chromosome position, the start and the end of ROHet islands and the corresponding lists of genes. The three ROHet islands were found on BTA6, BTA14 and BTA21, ranging in length from 0.43 MB (BTA06) to 0.79 MB (BTA21). The ROHet island on BTA6 was found to harbor three genes, those on BTA14 and BTA21, respectively, two and 12 genes.

### 3.6. ROH Sensitivity Analysis

**Figure 6:**
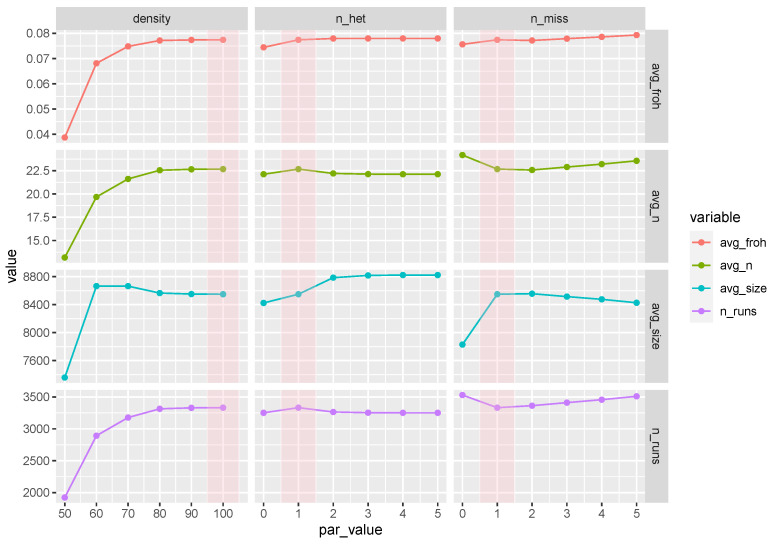
Number of ROH (n_runs), average ROH size (avg_size), average number of ROH per animal (avg_n) and average ROH-based inbreeding (avg_froh) per different values of parameters used to detect ROH in the Maremmana cattle genome: density (1 SNP every n kbps), number of heterozygous genotypes allowed in a ROH (n_het), number of missing genotypes allowed in a ROH (n_miss). The areas shaded in pink highlight the base scenario (density = 100, n_het = 1, n_miss = 1).

Results from the sensitivity of ROH detection to the parameters used for their identification are reported in Figure 6: the shaded areas highlight the parameter values used in the base ROH-detection scenario. By increasing density progressively from 1 SNP every 100 kB to 1 SNP every 50 kB, the number of detected ROH (total and per animal) and the average $F_{ROH}$ decrease approximately logarithmically (initially very slow, then progressively faster); the average size of detected ROH, however, remained rather constant in the range 8.55–8.65 MB down to 1 SNP every 60 kB, before plummeting abruptly to 7.4 MB for 1 SNP every 50 kB. Increasing the number of heterozygous or missing SNPs allowed in an ROH from 1 (base scenario) to 5 had overall a limited impact on the results from ROH detection: at most, a small increase in the number of detected ROH and in $F_{ROH}$. Conversely, not allowing any heterozygous or missing SNP in an ROH reduced the size of detected ROH and the average $F_{ROH}$, while the effect on the number of ROH (overall and per animal) was less clear.

## 4. Discussion

In this paper, we presented results from the genetic characterization of semi-feral Maremmana cattle: from pedigree and genomic data the basic genetic diversity parameters have been estimated, and properties of specific segments of the genome have been described in terms of runs of homozygosity and heterozygosity-rich regions. Specific aspects are discussed here in detail: genetic diversity of Maremmana cattle, distribution of ROH and ROHet, genes linked to islands of homozygosity and heterozygosity, and the methodological implications for semi-feral cattle populations.

### 4.1. Genetic Diversity of Maremmana Cattle

**Figure 7:**
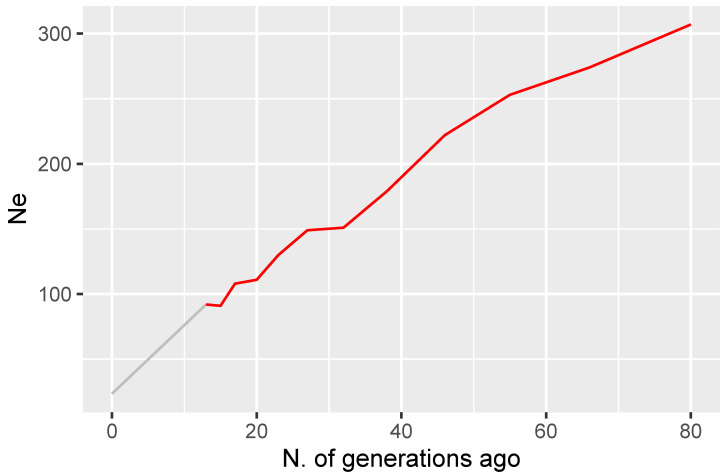
Historical (from SNeP) and current (from Neestimator) estimates of effective population size (Ne) in Maremmana cattle.

An estimated Ne of 23.5 was obtained here from SNP genotypes; larger Ne estimates were obtained in previous studies on Maremmana cattle from large pedigree data: 110.9 [34] and 123.5 [35]. Besides the different methodologies based on genealogies rather than genomics, the observed differences in the estimated Ne can be attributed to the different timeframes: pedigree records from 2001 [34] and average Ne over the period 1980–2015 [35]. To correctly place our point-estimate of Ne in perspective, we estimated historic Ne based on the relationship between LD ($r^{2}$), Ne and recombination rate [36], as implemented in the SNeP tool [37]: results (Figure 7) show a declining Ne trend over time, with values between 307 and 23.5 in the last 80 generations, which is consistent with the trajectory of Ne in most non-African taurine breeds [38].

The estimates of $H_{E}$ and $H_{O}$ were not dissimilar, 0.261 and 0.274 respectively, which suggests that there are no major recent events pushing heterozygosity in either direction. On a smaller sample size (N=12), $H_{E}$ were estimated at 0.369 and 0.381 from 150k SNP data [39]. More recently, from 48 k SNP data and sample size N=25, estimates of $H_{E}=0.319$ and $H_{O}=0.338$ were obtained [40]. Previous studies based on microsatellite or amplified fragment-length polymorphism (AFLP) markers gave more variable results for heterozygosity, in the range 0.14–0.73 [41,42,43]. The heterozygosity estimated in Maremmana cattle is at intermediate values among cattle breeds [38].

Genetic relationships between individual animals were calculated from genealogies and from SNP data, either from SNP genotypes directly or from ROH. The substructure linked to the two herds is very apparent in genealogy-based relationships (Figure 1), probably because of the few connections recorded in the rather shallow and partially complete available pedigree file. The use of in-herd dams for natural mating may also have contributed. This substructure is much less clear in genomic relationships, either from single SNP or ROH, which evidently are able to capture unrecorded and deeper genetic connections between animals.

From genetic relationships, inbreeding levels can be estimated. Pedigree-based average inbreeding was estimated at 4.9%; other works estimated pedigree inbreeding at 6.76% [34], 7.1% [44] and 2.78% [35]. Besides different pedigree size, these differences may also reflect the different timeframes when inbreeding was estimated. The estimation of the inbreeding coefficient *F* has traditionally relied on deep pedigree records which included relationships between remote ancestors; however, in most cases this is unavailable or inaccurate, especially for semi-feral local breeds. In addition, the probabilistic approach of pedigree analysis does not take into account the stochastic nature of recombination [10]. SNP genotypes offer a valid alternative that is both accurate and practical, used either as single-locus genotypes or as haplotypes like ROH. Alongside *F*, $F_{IS}$ and $F_{ROH}$ have been estimated. The correlation was low between *F* and $F_{IS}$ or between *F* and $F_{ROH}$, while it was high between $F_{IS}$ and $F_{ROH}$. High correlation between $F_{IS}$ and $F_{ROH}$ (0.84) was also reported in pigs [45] and other cattle breeds [7,9].

The comparison of *F* and $F_{ROH}$ revealed a poor correlation in Maremmana cattle, as reported in other cattle breeds (e.g., [6,28]). On the other hand, a strong correlation between pedigree-based and ROH-based inbreeding estimates has been reported by other authors (e.g., [8,9]). An important factor in such studies has been pedigree depth, and generally the strongest correlations were found for animals with the largest number of recorded generations. Therefore, the relatively low correlation found in this study may have resulted from the limited pedigree depth available for these animals. The results of this study confirm that $F_{ROH}$ may be a useful estimator of individual autozygosity in cattle populations, and may give some insights on pedigree-based inbreeding estimates in cases when historic pedigree records are unavailable.

### 4.2. Runs of Homozygosity and Heterozygosity-Rich Regions

Runs of homozygosity have been characterized in many cattle populations, not yet in the Maremmana breed. In the present study, 3332 ROH longer than 2 MB have been detected in the genome of Maremmana cattle, with an average of 22.27 ROH per animal. The genome of Maremmana cattle comprises mostly a high number of short segments (2–4 MB), which accounted for 36.2% of all ROH detected. In general, the comparison of ROH results is not straightforward since different studies used different criteria. Szmatoła et al. [46] reported notable differences in the length and amount of ROH between local breeds and commercial breeds, with an average number of ROH per animal in the range from 21 to 30 for local breeds, in agreement with our results. In addition, as identified here in the Maremmana breed, local breeds were characterized on average by few ROH longer than 8 MB compared to commercial breeds [7,8,46,47].

Clustering ROH into different size classes makes it possible to interpret patterns of homozygosity. Our study revealed that in the Maremmana breed most ROH are short or middle-size. The expected length of autozygous segments that are IBD follows an exponential distribution with mean equal to 0.5 g Morgans, where *g* is the number of generations since the common ancestor [48]: 16 MB ROH segments are expected to reflect inbreeding up to three generations in the past, while short ROH ( 4 MB) are related to more ancient inbreeding, up to 12.5 generation in the past (about 75 years in cattle). Our findings, therefore, suggest that Maremmana cattle experienced both recent and ancient inbreeding events, given that some animals lacked longer ROH, while others showed long segments. Similar results for $F_{ROH>2MB}$ were reported by Ferenčaković et al. [9] using a 50K panel for Norwegian Red (0.074) and Tyrol Grey (0.069) local cattle breeds.

Heterozygosity-rich regions are far less characterized than ROH in livestock. Williams et al. [16] were the first to introduce the concept of ROHet in diploid genomes, and used the highly homozygous semi-feral Chillingham cattle population as a model: they observed that heterozygosity loci were clustered in islands along the genome, and reported an average of 9.5 ROHet ≥150 kB per chromosome. Ferenčaković et al. [49] reported an average of 121.5 ROHet per animal in Pinzgauer cattle. Outside of cattle, Marras et al. [50] detected 57.8 ROHet per animal with an average length of 470 kB in commercial turkeys; dos Santos et al. [17] found 52.2 ROHet per animal with average length of 80 kB in a local horse breed. Differences with our results may be attributed to different methods, animal population and SNP array. In spite of this heterogeneity of methodology and data, all studies found that heterozygosity-regions were much rarer and shorter compared to ROH.

It is known that the parameters used to detect ROH influence results and vary widely across studies [26]. There is still no systematic review of the parameters for ROH identification, and only a few studies attempted to look at the effect of tweaking some specific parameters. Ferenčaković et al. [51] and Mastrangelo et al. [7] found that allowing heterozygous SNPs in ROH to account for genotyping errors leads to higher estimates of $F_{\mathrm{ROH}}$. Forutan et al. [52] looked at the size of the sliding window and reported that the smaller the window the higher the $F_{ROH}$. In our study, we varied the number of heterozygous and missing SNP allowed within ROH (from 0 to 5), and did not observe a dramatic effect on the number of ROH detected, on their size and on $F_{\mathrm{ROH}}$, except for the most stringent scenario where no heterozygous nor missing SNPs were allowed inside ROH. In this latter case, fewer and shorter ROH were detected and, consequently, a lower $F_{\mathrm{ROH}}$ was estimated. On the other hand, with increasing numbers of heterozygous and missing SNPs the number of ROH detected, their size and $F_{\mathrm{ROH}}$ increased only slightly. The parameter with the largest bearing on results is SNP density: when a higher SNP density is required, fewer and shorter ROH are detected, and a smaller $F_{\mathrm{ROH}}$ is estimated. The relationship is approximately logarithmic, with results tending to plateau as SNP density is relaxed (Figure 6). These results relate to findings on the effect of SNP array density previously reported in literature [8,51].

**Figure 8:**
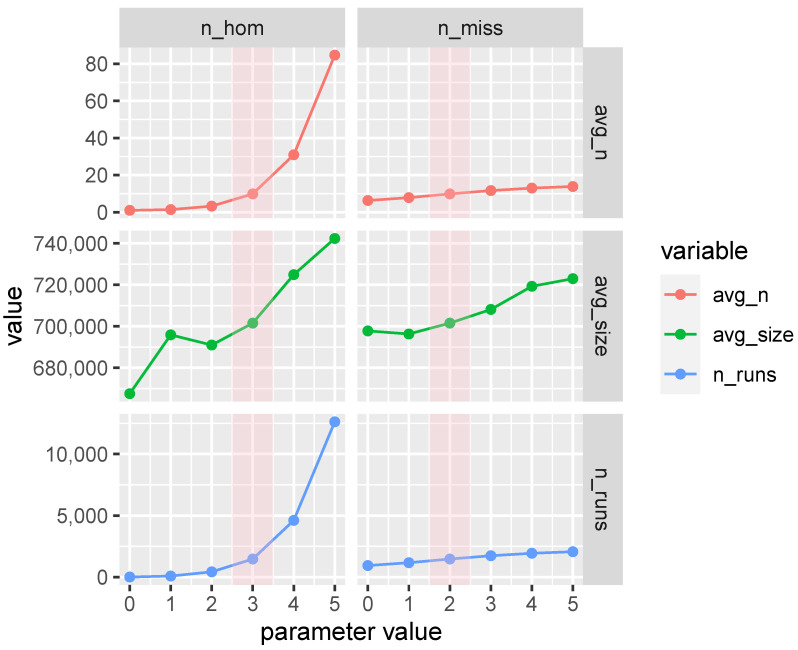
Number of ROHet (n_runs), average ROHet size (avg_size) and average number of ROHet per animal (avg_n) per different values of parameters used to detect ROHet in the Maremmana cattle genome: number of homozygous genotypes allowed in a ROHet (n_hom), number of missing genotypes allowed in a ROHet (n_miss). The areas shaded in pink highlight the base scenario (n_hom = 3, n_miss = 2).

Nothing has been reported so far on the effect of tweaking parameters for the detection of heterozygosity-rich regions (ROHet): in Figure 8 we present the results of the first sensitivity analysis for ROHet. We varied the number of missing and homozygous SNPs allowed inside ROHet: contrary to ROH, we observed a sharp increase in the number of ROHet detected and in their average size when increasing numbers of missing and/or homozygous SNPs are allowed. This different behavior between ROHet and ROH may be due to the different nature of ROHet: these latter are not “runs” stricto sensu, rather heterozygosity-rich regions in which interspersed homozygous loci are expected to be found beyond genotyping errors [49].

### 4.3. Biological Meaning of ROH-Islands and Islands of Heterozygosity

ROH islands in cattle have been used to identify genomic regions under selection, which may be involved in defining breed-specific traits or the adaptation to the environment or production systems [14,53,54]. The ROH islands identified in the Maremmana breed are linked to a number of candidate genes involved in several biological functions. The *KCTD8* gene is associated with climate adaptation [20] and has been reported within selection signatures in cattle [55]. This gene, together with *YIPF7*, *GUF1*, *GNPDA2*, *GABRG1* and *GABRA2* have been associated with carcass traits (rib-eye area) in a composite beef cattle breed [56]. *GNPDA2* has been found to have a potential role in the regulation of body weight and of fat and energy metabolism in chickens [57].

Unlike ROH islands, heterozygosity-rich regions have not been analysed much in the genome of livestock populations. Using a high-density SNP array Williams et al. [16] found that although ROH segments covered about 90% of the genome of Chillingham cattle, some regions were highly heterozygous. These “Runs of Heterozygosity” (ROHet) can harbor loci that contribute to survival rate, fertility and other fitness-related traits [58], and are probably segments of the genome where diversity can be very beneficial. The relationship between genetic diversity and fitness is an important topic in evolutionary biology: for instance, ROHet islands could point to events of balancing selection [59]. In Maremmana cattle, within the ROHet hotspots several known genes have been identified (Table 3). For the *GABRB1* gene on BTA6 in Maremmana cattle, Hou et al. [60] reported an association with nematode resistance. On BTA21, there are four genes (*TARSL2*, *TM2D3*, *PCSK6* and *SNRPA1*) related to reproduction traits in livestock [61]. *PCSK6* (involved in iron homeostasis) may be associated with the evolutionary response to anemia in Sheko cattle [62]. In those genomic regions that harbor loci involved in general disease resistance, a high level of genetic diversity (“ROHet hotspots”) ensures that the population can deal with potential new disease challenges.

## 5. Conclusions

This is the first exhaustive genome-wide study of the genetic diversity in semi-feral Maremmana cattle using both pedigree- and genomic-based data. Estimators of inbreeding based on different data types and statistical approaches were compared. The correlation between pedigree-based and genome-based inbreeding was low, plausibly due to the limited depth of the available pedigree data for these animals. The genetic diversity parameters and the patterns of ROH found in Maremmana cattle were in agreement with results from previous studies in local cattle breeds. Genes found to be within the identified ROH and ROHet islands point to phenotypic characteristics related to environmental adaptation and robustness of Maremmana cattle. Taken together, these results indicate that genome-based measures of inbreeding and heterozygosity through ROH and ROHet are able to detect differences between chromosomal regions, providing a more detailed picture of the genetic diversity. The results are of significant importance because they will help design and implement breeding and conservation strategies for Maremmana cattle.

## Figures and Tables

**Table 1:** Descriptive statistics of ROH by size classes.

Class (MB)	N.	Avg. Size (kB)	Freq.
2–4	1206	2998	0.36
4–8	1053	5607	0.32
8–12	416	9737	0.12
12–16	211	13,852	0.06
16–20	153	17,786	0.05
20–30	162	24,085	0.05
>30	131	40,972	0.04

**Table 2:** Descriptive statistics of heterozygosity-rich regions (ROHet) by size classes.

Class (kB)	N.	Avg. Size (kB)	Freq.
250–500	165	451	0.11
500–750	821	617	0.56
750–1000	372	850	0.25
1000–1500	106	1141	0.07
1500–2000	5	1744	0.00
>2000	2	2071	0.00

**Table 3:** ROH and ROHet islands detected in Maremmana cattle. Non-annotated loci are indicated with the prefix LOC.

	BTA	Start (bp)	End (bp)	N SNPs	Genes
ROH islands	6	61,877,121	64,865,529	58	LOC112447156, LOC112447157, LOC101906152, TRNAC-ACA, KCTD8, YIPF7, GUF1, GNPDA2, TRNAC-ACA, LOC112447181, LOC112447015, GABRG1, GABRA2
	6	65,343,631	65,774,333	15	COX7B2, GABRA4, GABRB1
ROHet islands	14	51,026,484	51,596,222	16	LOC112449555, LOC112449527
	21	28,714,736	29,509,054	18	LOC104975368, TARSL2, TM2D3, PCSK6, LOC101906554, TRNAC-GCA, LOC100300380, SNRPA1, LOC100848886, LOC789895, LOC112443140, LOC112443139
